# Central Sleep Apnea in Patients due to Severe Aortic Stenosis

**DOI:** 10.1155/2010/952634

**Published:** 2010-06-14

**Authors:** Christian Prinz, Thomas Bitter, Olaf Oldenburg, Lothar Faber, Dieter Horstkotte, Cornelia Piper

**Affiliations:** Department of Cardiology, Heart and Diabetes Center North Rhine-Westphalia, Ruhr University Bochum, Georgstraße 11, 32545 Bad Oeynhausen, Germany

## Abstract

*History*. We report about the course of central sleep apnea (CSA) in 3 patients (70.3 ± 15.2 years) with severe aortic stenosis (AS) (AVA ≤ 1.0 cm^2^, NYHA 2.7 ± 1.4). *Investigations*. Every patient received echocardiography, left/right-heart catheterization, and cardiorespiratory polygraphy before and 6 months after surgical aortic valve replacement (without right-heart catheterization during follow up). *Course*. Preoperatively all patients demonstrated reduced systolic left ventricular function (EF <55%). They had elevated pulmoraryarterialy pressures and severe CSA. After valve replacement left ventricular function and exercise capacity improved, as well as the severity of CSA. *Conclusion*. Patients with severe AS can develop CSA, which seems to improve after surgery. Patients with severe AS should be screened for CSA, because CSA might be an additional risk factor and hint that myocardial adaptation is exhausting.

## 1. Introduction

Sleep disordered breathing (SDB) has a prognostic impact on cardiac patients [1]. Development of CSA, particularly Cheyne-Stokes respiration, because of secondary pulmonary hypertension due to reduced left ventricular function is known [2]. Advanced valvular heart disease with secondary pulmonary hypertension due to impaired diastolic and/or systolic function of the left ventricle may support the occurrence of CSA. We examined three patients with aortic stenosis (AS) before and after surgical valve replacement for CSA.

## 2. Case Presentation

### 2.1. Case 1

Patient 1 reported about angina pectoris for 1-2 years (CCS II-III) and progredient dyspnea. Echocardiography revealed severe AS (AVA 0.8 cm², mean gradient 35 mmHg). Left ventricular systolic function was reduced to an ejection fraction of 50%. By heart catheterization an additional two-vessel coronary artery disease (CAD) was diagnosed. 

### 2.2. Case 2

Patient 2 suffered from increasing dyspnea (NYHA III). Echocardiography identified severe AS (AVA 1.0 cm², mean gradient 25 mmHg). Left ventricular ejection fraction was reduced to 40%. CAD was excluded by catheterization.

### 2.3. Case 3

Patient 3 reported about moderate dyspnea (NYHA II). Echocardiography demonstrated severe AS (0.7 cm², mean gradient 44 mmHg). By catheterization a three-vessel CAD was found. Left ventricular ejection fraction was low with 22%. 

All sleep apnea examinations were done with a mobile device during hospital stay (Embletta, Embla, Amsterdam). A standard classification was used for description of SDB [3]. If thoracic and abdominal inspiration efforts were documented, SDB was considered to be obstructive (OSA), otherwise central sleep apnea (CSA) was diagnosed. If both, central and obstructive events were observed, OSA or CSA was diagnosed according to the majority of either events (cut off 50% of central versus obstructive events). Patients with an apnea-hypopnea-index (AHI) > 5/h were considered to have SDB. An AHI ≥ 15/h indicates moderate SDB and necessity for therapy [3]. Continuous data are expressed as mean value ± standard deviation (SD). Statistical analyses were performed with the Statview software (SAS Corporation). For continuous and normally distributed data, unpaired *t*-tests, in case of non-normality of distribution Wilcoxon-signed rank tests were used. A two-tailed *P* value < .05 was considered significant.

**Figure 1:**
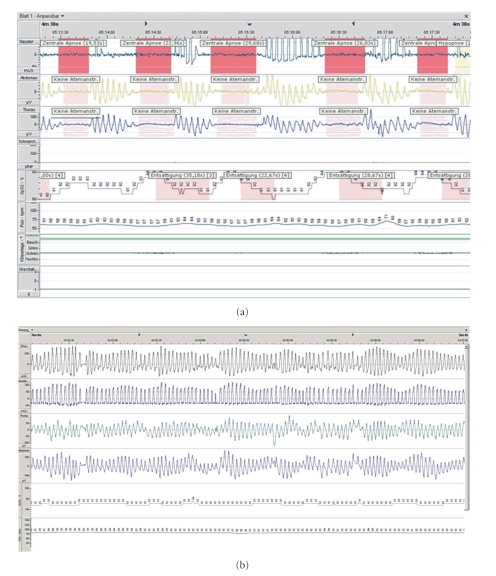
Respiratory analysis of a patient before (a) and after (b) aortic valve replacement. (a) Typical breathing in CSA without respiration efforts (nasal airflow, abdominal and thoracic breathing efforts, oxygen saturation, and heart rate are shown), (b) Only mild undulating nocturnal respiration.

Before valve replacement all patients suffered from severe CSA and had a mean AHI of 34 ± 41.5 /h (Table 1). Mean pulmonary artery pressure was elevated to 36.7 ± 22.5 mmHg. Six months after valve replacement in all patients the AHI-reduced significantly (mean AHI 6.3 ± 10.3 /h, *P* < .05) (Figure 1). Patient 3 still demonstrated a mild OSA. Pulmonary artery pressure after valve replacement was calculated by CW-Doppler of the regurgitation jet of the tricuspid valve and was also decreased after surgery (21.7 ± 7.2 mmHg, *P* = .05). After valve replacement left ventricular ejection fraction improved considerably (mean LVEF 39% versus 55%; *P* = ns) and was associated with better exercise capacity (NYHA: 2.7 ± 1.4 versus 0.7 ± 1.4; *P* = .01).

## 3. Discussion

Recent studies demonstrated that occurrence and severity of CSA are correlated to the severity of systolic and diastolic left ventricular dysfunction [4]. The occurrence of CSA is partially caused by secondary pulmonary hypertension following diastolic and/or systolic left ventricular dysfunction [2]. In patients with severe valve disease, secondary pulmonary hypertension due to exhaustion of myocardial left ventricular function may induce CSA. 

We report about three patients with severe AS and CSA before surgical valve replacement. Patient 1 had the best systolic left ventricular function and demonstrated the lowest severity of CSA. Before valve replacement all patients had increased mean pulmonary artery and left end diastolic pressure.

After valve replacement left ventricular function had been normalized in all three patients. Echocardiography revealed normal end diastolic and end systolic diameters and a decrease of left ventricular hypertrophy in all patients. All patients recovered from CSA postoperatively.

The relation between occurrence and severity of CSA to occurrence and severity of pulmonary hypertension must be verified in further studies. CSA was decreased or resolved after improvement in cardiac function. This supports that CSA is another manifestation of cardiac dysfunction. Therefore, asymptomatic patients with moderate to severe AS should be screened for the presence of CSA. Yet it is unclear, if the development of CSA might be an early and sensitive marker to detect myocardial maladaptation in patients with so-far asymptomatic AS. Further controlled studies with pre- and postoperative evaluations are warranted.

## Figures and Tables

**Table 1:** Clinical, hemodynamic, echocardiographic, and sleep data before and after aortic valve replacement (AVR). BMI: body mass index, AVA: aortic valve area, EF: ejection fraction, LVID(d/s): left ventricular diameter (end diastolic/end systolic), LVPWd: left ventricular posterior wall thickness, PAM: mean pulmonary artery pressure, LVEDP: left ventricular enddiastolic pressure, SDB: sleep disordered breathing, AHI: apnea/hypopnea-index.

	patient 1 before AVR	patient 2 before AVR	patient 3 before AVR	patient 1 after AVR	patient 2 after AVR	patient 3 after AVR	*P*
BMI (kg/m²]	26,7	28,4	23,5	27,3	27,4	26,8	ns
AVA [cm²]	0,8	1,0	0,7	2,1	2,3	1,7	*P* < .05
Mean Gradient [mmHg]	35	25	44	9	6	20	*P* < .05
EF [%]	55	40	22	55	55	55	ns
LVIDd [mm]	49	51	72	48	49	54	ns
LVIDs [mm]	25	34	60	16	34	34	ns
LVPWd [mm]	13	10	12	9	9	12	ns
PAM [mmHg]	30	33	47	20 (Echo)	25 (Echo)	20 (Echo)	*P* = .05
LVEDP [mmHg]	45	32	39				
SDBAHI [/h]	CSA16	CSA37	CSA49	None <5	None <5	OSA11	*P* < .05
